# HIV-1-specific CD8^+^ T cells with different abilities to recognize HIV-1-infected cells in HIV-1-exposed seronegative individuals

**DOI:** 10.1093/pnasnexus/pgaf336

**Published:** 2025-10-22

**Authors:** Hung The Nguyen, Takayuki Chikata, Nozomi Kuse, Yu Zhang, Diep Thi Ngoc Pham, Nga Thi Do, Thanh Cong Nguyen, Ngoc Bich Lung, Hao Thi Minh Bui, Hiroyuki Gatanaga, Giang Van Tran, Binh Thanh Nguyen, Do Van Nguyen, Le Minh Giang, Shinichi Oka, Masafumi Takiguchi

**Affiliations:** Division of International Collaboration Research and Tokyo Joint Laboratory, Joint Research Center for Human Retrovirus Infection, Kumamoto University, 2-2-1 Honjo, Chuo-ku, Kumamoto 860-0811, Japan; Division of International Collaboration Research and Tokyo Joint Laboratory, Joint Research Center for Human Retrovirus Infection, Kumamoto University, 2-2-1 Honjo, Chuo-ku, Kumamoto 860-0811, Japan; AIDS Clinical Center, National Center for Global Health and Medicine, Japan Institute for Health Security, Tokyo 162-8655, Japan; Division of International Collaboration Research and Tokyo Joint Laboratory, Joint Research Center for Human Retrovirus Infection, Kumamoto University, 2-2-1 Honjo, Chuo-ku, Kumamoto 860-0811, Japan; Division of International Collaboration Research and Tokyo Joint Laboratory, Joint Research Center for Human Retrovirus Infection, Kumamoto University, 2-2-1 Honjo, Chuo-ku, Kumamoto 860-0811, Japan; Department of Pathophysiology and Immunology, Hanoi Medical University, Hanoi 10000, Vietnam; Department of Pathophysiology and Immunology, Hanoi Medical University, Hanoi 10000, Vietnam; Center for Training and Research on Substance Abuse and HIV, Hanoi Medical University, Hanoi 10000, Vietnam; Center for Training and Research on Substance Abuse and HIV, Hanoi Medical University, Hanoi 10000, Vietnam; Center for Training and Research on Substance Abuse and HIV, Hanoi Medical University, Hanoi 10000, Vietnam; AIDS Clinical Center, National Center for Global Health and Medicine, Japan Institute for Health Security, Tokyo 162-8655, Japan; Department of General Planning, National Hospital of Tropical Diseases, Hanoi 10000, Vietnam; Department of Pathophysiology and Immunology, Hanoi Medical University, Hanoi 10000, Vietnam; Department of Pathophysiology and Immunology, Hanoi Medical University, Hanoi 10000, Vietnam; Dinh Tien Hoang Institute of Medicine, Hanoi 10000, Vietnam; Center for Training and Research on Substance Abuse and HIV, Hanoi Medical University, Hanoi 10000, Vietnam; School of Preventive Medicine and Public Health, Hanoi Medical University, Hanoi 10000, Vietnam; AIDS Clinical Center, National Center for Global Health and Medicine, Japan Institute for Health Security, Tokyo 162-8655, Japan; Division of International Collaboration Research and Tokyo Joint Laboratory, Joint Research Center for Human Retrovirus Infection, Kumamoto University, 2-2-1 Honjo, Chuo-ku, Kumamoto 860-0811, Japan

**Keywords:** HIV-1, T cells, CD8, HESN, MSM

## Abstract

The presence of HIV-1-specific CD8^+^ T-cell responses in HIV-1-exposed seronegative (HESN) individuals has been reported, but the details of these T cells have yet to be analyzed. We investigated HIV-1-specific CD8^+^ T-cell responses to 281 17-mer overlapping HIV-1 peptides and six HLA-B*15:02-restricted HIV-1 subtype AE epitope peptides in 370 Vietnamese HESN men who have sex with men (MSM). Analysis of cultured T cells stimulated with these peptides using intracellular cytokine staining (ICS) assay demonstrated HIV-1-specific CD8^+^ T-cell responses in only eight of these HESN-MSM. HLA-restricted CD8^+^ T cells specific for five HIV-1 epitope peptides were identified in five of these individuals by ICS assay and/or assay using HLA multimers. CD8^+^ T cells specific for three HIV-1 peptides (GagHL9, PolSV9, and PolVF9) recognized HIV-1 subtype AE–infected cells, whereas those specific for two HIV-1 peptides did not recognize them. Among CD8^+^ T cells that can recognize HIV-1-infected cells, those specific for two epitopes, GagHL9 and PolSV9, were frequently elicited in HIV-1-infected individuals, whereas PolVF9-specific CD8^+^ T cells were not found in them. These results indicate that two types of HIV-1-specific CD8^+^ T cells were clearly detected in HESN-MSM; they include CD8^+^ T cells specific for the immunodominant or nonimmunodominant HIV-1 epitope peptide that is effectively presented in HIV-1-infected cells and those for the HIV-1 peptide that is very weakly or not presented by HLA class I in HIV-1-infected cells. The present study demonstrated the existence of different HIV-1-specific CD8^+^ T cells that can or cannot recognize HIV-1-infected cells before HIV-1 infection is established.

Significance Statement The existence of HIV-1-exposed seronegative (HESN) individuals suggests a mechanism of natural resistance to HIV infection. Previous studies showed HIV-1-specific CD8^+^ T-cell responses in some HESN individuals, suggesting that T-cell-mediated immunity contributes to reducing HIV-1 infection risk. However, the features and functions of these T cells remain poorly understood. Our study demonstrated the presence of two types of CD8^+^ T cells in MESN individuals. They are T cells specific for the immunodominant or nonimmunodominant HIV-1 epitope that is effectively presented in HIV-1-infected cells and for an HIV-1 peptide that is very weakly or not presented in HIV-1-infected cells. The present study clarified some of the features of CD8^+^ T-cell immunity against HIV-1 before HIV-1 infection.

## Introduction

Some individuals remain uninfected despite repeated exposures to people living with HIV-1 in couples with HIV-1 seronegative discordance, commercial sex workers with unprotected sexual contact, and men who have sex with men (MSM) within HIV-1 endemic regions (1–4). T-cell-mediated immunity and innate immunity may protect against HIV-1 infection in HIV-1-exposed seronegative (HESN) individuals. Indeed, many previous studies detected HIV-1-specific T-cell responses in some HESN individuals (5–20). However, it has yet to be determined whether these HIV-1-specific T-cell responses contribute to protecting against HIV-1 infection. A previous study showed that the number of Nef/Integrase/Vif-specific T cells in persistently HESN individuals was higher than that in preinfected samples from seroconverted individuals, suggesting that these T cells may contribute to lowering the risk of HIV-1 infection (19). Although some of the HIV-1 epitopes recognized by these T cells were identified, the ability of the T cells to recognize HIV-1-infected cells was not analyzed. T-cell responses to HIV-1 peptides in peripheral blood mononuclear cells (PBMCs) from HESN individuals were low in magnitude and breadth (11, 20–24), and transiently detectable (25, 26). Only a few studies have successfully established robust T-cell lines specific for HIV-1 epitope peptides (7, 11, 17, 27). The ability of these previously reported HIV-1-specific T cells to recognize HIV-1-infected cells was not investigated. Thus, whether HIV-1-specific CD8^+^ T cells in HESN individuals are able to recognize HIV-1-infected cells remains uncertain, and the features and functions of HIV-1-specific CD8^+^ T cells identified in HESN individuals are only partially understood.

It is speculated that HIV-1-specific CD8^+^ T cells are elicited in two possible ways in HESN individuals. One is that macrophages take up HIV-1 proteins and/or HIV-1-infected cells in the mucosal membrane and then present HIV-1 epitopes to CD8^+^ T cells via a nonclassical pathway. The other is that a small number of HIV-1-infected cells present epitopes to CD8^+^ T cells in the mucosal membrane via the classical pathway, while such HIV-1-infected cells might be eradicated by innate immunity such as NK cells. The mechanism for the induction of HIV-1-specific CD8^+^ T cells in HESN individuals and the details of such HIV-1-specific CD8^+^ T cells remain unknown. The study of HIV-1-specific CD8^+^ T cells in HESN individuals is valuable for understanding not only the features of HIV-1-specific CD8^+^ T cells before the establishment of HIV-1 infection but also the defense mechanism against HIV-1. In addition, such study should contribute to the development of a T-cell-targeting AIDS vaccine.

In the present study, we analyzed the presence of HIV-1-specific CD8^+^ T cells in 370 HESN-MSM in Vietnam. We attempted to detect HIV-1-specific CD8^+^ T cells in PBMCs from HESN-MSM by using an intracellular cytokine staining (ICS) assay after PBMCs from these individuals had been stimulated with HIV-1 peptides and then cultured for 3 weeks. We not only characterized the epitopes of HIV-1-specific CD8^+^ T cells elicited in HESN-MSM but also investigated the ability of these CD8^+^ T cells to recognize HIV-1-infected cells. We demonstrated that three types of HIV-1-specific CD8^+^ T cells were elicited in HESN-MSM.

## Results

### Detection of HIV-1-specific T cells in HESN-MSM

**Fig. 1.**
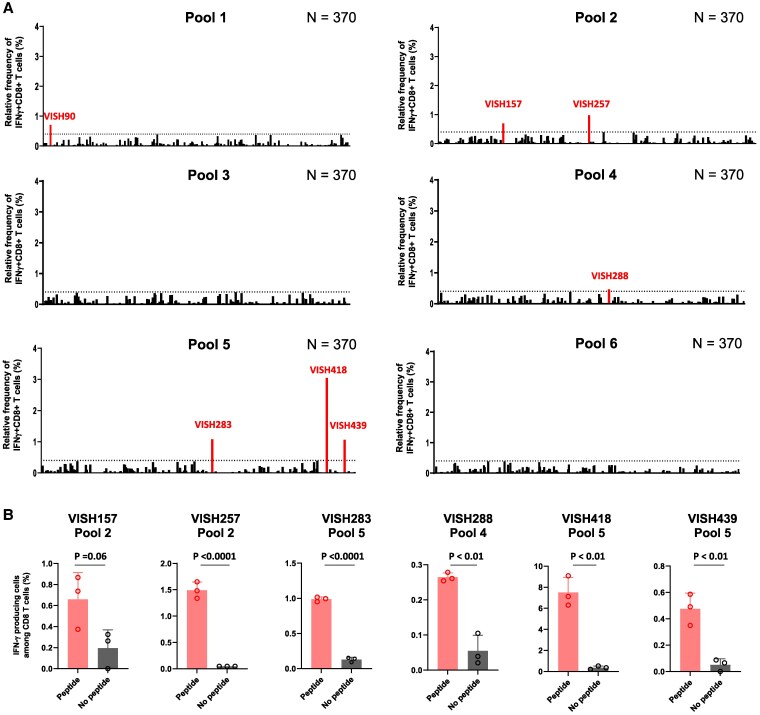
Detection of HIV-1-specific CD8^+^ T cells in 370 HESN-MSM. A) T-cell responses to six peptide pools in 370 HESN-MSM. T-cell responses in T cells cultured for 3 weeks were analyzed by ICS assay. The relative frequency of IFN-γ^+^CD8^+^ T cells was calculated as the frequency of IFN-γ-producing cells among CD8^+^ T cells stimulated with peptide pool minus the frequency of IFN-γ-producing cells among CD8^+^ T cells not stimulated with peptide pool. A dashed line shows the threshold of positive responses defined as a frequency of IFN-γ^+^CD8^+^ T cells of >0.4% relative to the total CD8^+^ T cells. Positive CD8^+^ T-cell responses are marked with a red bar. B) Positive responses to peptide pool in six HESN-MSM were confirmed by ICS assay in triplicate samples. Raw data of flowcytometric analysis is shown in Fig. S1. Statistical analysis was performed by unpaired t test.

We recruited 370 HESN-MSM Vietnamese and collected peripheral blood from them to analyze HIV-1-specific CD8^+^ T cells among HESN-MSM individuals in Vietnam, where HIV-1 subtype AE is prevalent. PBMCs from these individuals were stimulated with HIV-1 peptides and then cultured for 3 weeks. The cultured cells were analyzed to detect HIV-1-specific CD8^+^ T cells by performing ICS assay. For this analysis, we used 281 17-mer peptides covering Nef, Gag, and Pol and divided them into 35 peptide cocktails (8 or 9 17-mer peptides in each peptide cocktail) and into six peptide pools (see Materials and methods). Approximately 5 × 10^5^ PBMCs were stimulated with each peptide pool, followed by culture for 3 weeks. To detect T-cell responses to the corresponding peptide pool, the cultured cells were analyzed by an interferon-gamma (IFN-γ) ICS assay. To evaluate positive T-cell responses to peptide pool in the ICS assay at the first screening, we used the following criteria: (1) IFN-γ-producing cells constituted >0.4% of CD8^+^ T cells in samples stimulated with peptide pool and (2) IFN-γ-producing cells constituted <0.5% of CD8^+^ T cells in samples without peptide pool in the ICS assay. When positive responses to pooled peptides were identified, positivity was confirmed in an ICS assay of triplicate samples. Finally, we confirmed HIV-1-specific responses by analyzing responses to peptide cocktails in the peptide pool. From a large-scale analysis of this kind, we found seven positive responses to four peptide pools (pool 1, pool 2, pool 4, and pool 5) in 7 of 370 individuals in ICS assays of a single sample (Fig. 1A) and confirmed six positive responses in ICS assays of triplicate samples in the six individuals (Figs. 1B and S1).

We could not test the response to pool 1 in a triplicate assay for VISH90 because of the limited number of cultured cells, but further investigated this response because the response to pool 1 peptide was strong.

### Identification of HIV-1 epitopes recognized by HIV-1-specific CD8^+^ T cells in HESN-MSM

We first sought to identify single 17-mer peptides recognized by CD8^+^ T cells that responded to peptide pool in seven individuals. CD8^+^ T cells from these individuals recognized Nef3 cocktail (VISH90), Gag1 cocktail (VISH157 and VISH257), Pol4 cocktail (VISH288), Pol12 cocktail (VISH283 and VISH418), or Pol9 cocktail (VISH439; Fig. S2A). We next analyzed the recognition of these T cells for 17-mer single peptides in these cocktails (Fig. S3). The CD8^+^ T cells from VISH90, VISH157, VISH283, and VISH288 recognized Nef17-18/Nef17-19, Gag17-5/Gag17-6, Pol17-89/Pol17-90, and Pol17-26/Pol17-27 peptides, respectively, while the CD8^+^ T cells from VISH257, VISH418, and VISH439 recognized Gag17-5, Pol17-95, and Pol17-70 peptide, respectively (Fig. S2B).

**Fig. 2.**
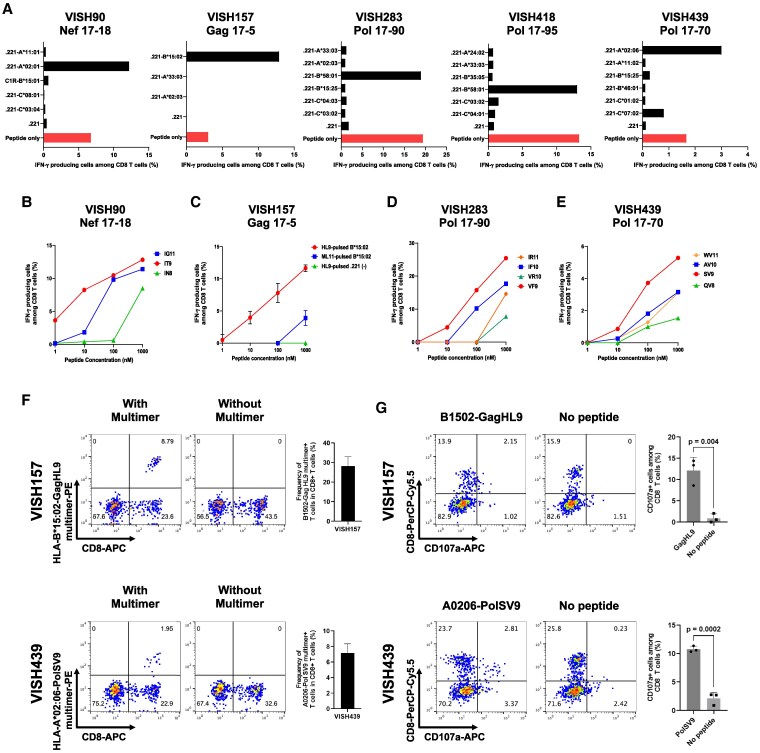
Identification of HIV-1 epitopes recognized by HIV-1-specific CD8^+^ T cells. A) Identification of HLA restriction of HIV-1-specific CD8^+^ T-cell responses. HLA class I alleles in five HESN-MSM were identified by HLA typing as follows: HLA-A*02:01/11:01, HLA-B*15:01/15:18, and HLA-C*03:04/08:01 (VISH90); HLA-A*02:03/33:03, HLA-B*15:02/55:02, and HLA-C*08:01/12:03 (VISH157); HLA-A*02:03/33:03, HLA-B*15:25/58:01, and HLA-C*03:02/04:03 (VISH283); HLA-A*24:02/33:03, HLA-B*35:05/58:01, and HLA-C*03:02/04:01 (VISH418); and HLA-A*02:06/11:02, HLA-B*15:25/46:01, and HLA-C*01:02/07:02 (VISH439). Responses of bulk T cells derived from five HESN-MSM to CD4.221 cells expressing a single HLA allele or C1R cells expressing HLA-B*15:01 and empty CD4.221 cells (.221) prepulsed with the 17-mer peptide at a concentration of 1 μM and at effector and target ratio of 1:5 were analyzed by ICS assay. IFN-γ-producing CD8^+^ T cells were also measured after bulk T cells were stimulated with the 17-mer peptide (peptide only). The name of 17-mer peptides used in each experiment is presented at a top of each figure. B) Responses of Nef17-18-specific CD8^+^ T cells derived from VISH90 to CD4.221-A*02:01 prepulsed with three truncated peptides at concentrations from 1 to 1,000 nM. C) Responses of Gag 17-5-specific CD8^+^ T cells derived from VISH157 to CD4.221-B*15:02 or CD4.221 cells prepulsed with GagHL9 or Gag ML11 at concentrations from 1 to 1,000 nM in triplicate assays. D) Responses of Pol17-90-specific CD8^+^ T cells derived from VISH283 to CD4.221-B*58:01 prepulsed with four truncated peptides at concentrations from 1 to 1,000 nM. E) Responses of Pol 17-70-specific CD8^+^ T cells from VISH439 to CD4.221-A*02:06 cells prepulsed with four truncated peptides at concentrations from 1 to 1,000 nM. F) Identification of GagHL9- and PolSV9-specific CD8^+^ T cells by using multimers of HLA-B*15:02-GagHL9 and HLA-A*02:06-PolSV9. Representative data (left) and analysis of triplicate samples (right) are shown. G) Expression of CD107a on GagHL9- and PolSV9-specific CD8^+^ T cells after stimulation with epitope peptides. Representative data (left) and analysis of triplicate samples (right) are shown. Statistical analysis was performed by unpaired t test.

We next sought to identify HLA restriction of these T-cell responses. We attempted to expand T cells specific for these 17-mer peptides for experiments identifying HLA restriction of the T-cell responses. However, the T cells from VISH257 and VISH288 were not expanded after stimulating these cultured cells with the corresponding 17-mer peptides. We therefore identified HLA restriction of the T cells from only five individuals by performing ICS assays using HLA class I–deficient 721.221 cell lines expressing CD4 molecules (CD4.221 cells) expressing a single HLA allele or C1R-B*15:01. We determined HLA class I alleles of these individuals. VISH157 had HLA-A*02:03/33:03, HLA-B*15:02/55:02, and HLA-C*08:01/12:03, but we neither had CD4.221 cells/C1R cells expressing single HLA-B*55:02, HLA-C*08:02, nor HLA-C*12:03. CD8^+^ T cells from this individual recognized Gag17-5 peptide including HLA-B*15:02-restricted epitope, GagHL9 (28). Therefore, we analyzed the T-cell response to Gag17-5 peptide by using CD4.221-B*15:02, CD4.221-A*02:03, and CD4.221-A*33:03. The bulk T cells recognizing Nef17-18, Gag17-5, Pol17-90, Pol17-95, or Pol17-70 were re-stimulated with each 17-mer peptide and cultured. These cultured bulk cells were used as effector T cells. The result showed that the T-cell response to Gag17-5 peptide is restricted by HLA-B*15:02 (Fig. 2A). The responses of T cells from other four individuals were analyzed by using the cell lines expressing six or five HLA alleles. The responses of CD8^+^ T cell from VISH90, VISH283, VISH418, and VISH439 were restricted by HLA-A*02:01, HLA-B*58:01, HLA-B*58:01, and HLA-A*02:06, respectively (Fig. 2A).

T cells specific for Pol17-95 did not expand in the cultured cells. Therefore, we sought to identify epitope peptides recognized by the other four T cells. First, we investigated which 11-mer peptides covering these 17-mers were recognized by these T cells. CD8^+^ T cells from VISH157 recognized both Gag17-5 and Gag17-6, but the former 17-mer more than the latter one, while those from VISH439 recognized only Pol17-70 (Fig. S2B). These results suggest that the epitopes were included in Gag17-5 and Pol17-70. We generated 11-mer peptides in these 17-mer ones and tested the recognition of these 11-mers by the corresponding T cells. CD8^+^ T cells from VISH157 recognized only GagML11, while those from VISH439 recognized only PolWV11 (Figs. S4B and S2D). We previously identified the HLA-B*15:02-restricted GagHL9 epitope in subtype AE–infected Vietnamese (28). Therefore, it was speculated that the epitope of HLA-B*15:02-restricted T cells from VISH157 is the GagHL9 epitope. Indeed, the T cells from VISH157 recognized GagHL9 more than GagML11 peptide (Fig. 2C).

CD8^+^ T cells from VISH90 and VISH283 recognized both Nef17-18 and Nef17-19 peptides and both Pol17-89 and Pol17-90 peptides at similar levels, respectively (Fig. S2B). We therefore speculated that the epitopes are included in overlapping parts, NefIG11 (ILDLWVYNTQG) and PolIR11 (IVIWGKTPKFR), between these two 17-mers. We generated truncated peptides of NefIG11, PolIR11, and PolWV11, after which we analyzed the recognition of the truncated peptides by the T cells from VISH90, VISH283, and VISH439. The CD8^+^ T cells from VISH90, VISH283, and VISH439 recognized NefIT9, PolVF9, and PolSV9, respectively, more than other truncated peptides and/or the 11-mer peptides (Figs. 2B and S4A, 2D and S4C, and 2E and S4D), indicating that these peptides were candidates of T-cell epitopes. The induction of NefIT9 (subtype B–specific sequence ILDLWVYHT)-specific T-cell responses by HIV-1 peptide-primed dendritic cells from two HIV-1-uninfected individuals was previously reported (29), while PolSV9 was reported as an HLA-A*02:06-restricted epitope in subtype B infection (30). In contrast, to the best of our knowledge, T-cell responses to PolVF9 were not reported.

We attempted to identify GagHL9-specific and PolSV9-specific CD8^+^ T cells by using multimers of HLA-B*15:02-GagHL9 and HLA-A*02:06-PolSV9, respectively. The specificity of these multimers was confirmed by using the specific T cells derived from HIV-1-infected individuals (Fig. S5). GagHL9- and PolSV9-specific CD8^+^ T cells were clearly demonstrated in the cultured cells from VISH157 and VISH439, respectively (Fig. 2F). We further analyzed whether these CD8^+^ T cells express CD107a after stimulation with epitope peptides. The results showed that GagHL9- and PolSV9-specific CD8^+^ T cells expressed CD107a after stimulation with GagHL9 and PolSV9 peptides, respectively (Fig. 2G), implying that these CD8^+^ T cells have killing function.

### Identification of HIV-1-specific CD8^+^ T cells by using reported HLA-B*15:02-restricted subtype AE epitope peptides in HESN-MSM

**Fig. 3.**
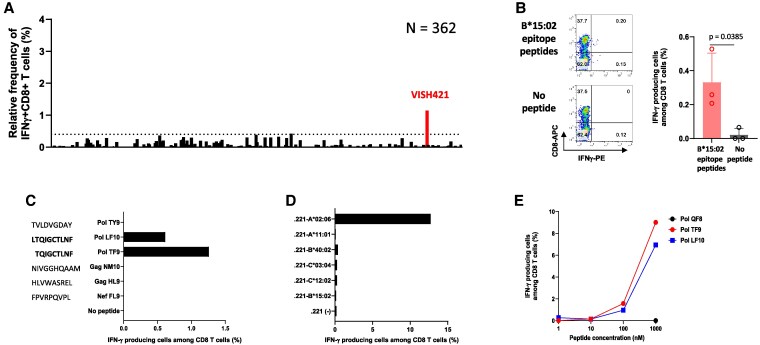
Identification of HIV-1-specific CD8^+^ T cells by using HLA-B*15:02-restricted epitope peptides. A) CD8^+^ T-cell responses to a cocktail of HLA-B*15:02-restricted epitope peptides were analyzed by ICS assay in 362 HESN-MSM. B) (Left) Representative data of CD8^+^ T-cell responses to a cocktail of HLA-B*15:02-restricted epitope peptides in VISH421. (Right) Responses of bulk T cells derived from VISH421 to a cocktail of HLA-B*15:02-restricted epitope peptides at a concentration of 1 μM were analyzed by ICS assay (*n* = 3). C) Responses of bulk T cells derived from VISH421 to each HLA-B*15:02-restricted epitope peptide (1 μM) were analyzed by ICS assay. D) Identification of HLA restriction of HIV-1-specific CD8^+^ T-cell responses. HLA alleles of VISH421 were identified by HLA typing as follows: HLA-A*02:06/A*11:01, B*27:04/40:02, and C*03:04/12:02. Responses of bulk T cells from VISH421 to CD4.221 cells expressing a single HLA or empty CD4.221 cells prepulsed with Pol TF9 at a concentration of 1 μM were analyzed by ICS assay. E) Responses of bulk T cells from VISH421 to CD4.221-A*02:06 prepulsed with PolLF10 and PolTF10 at concentrations from 1 to 1,000 nM were analyzed by ICS assay.

The number of reported CD8^+^ T-cell subtype AE epitope is very limited, but we recently identified six HLA-B*15:02-restricted AE epitopes in HIV-1 subtype AE–infected Vietnamese (28). Because HLA-B*15:02 is a relatively common allele among HLA-B in Vietnam, we attempted to identify CD8^+^ T cells specific for HLA-B*15:02 epitopes by using these reported HLA-B*15:02-restricted epitope peptides. We stimulated PBMCs from 362 HESN-MSM with a cocktail of six B*15:02-restricted subtype AE epitope peptides (Nef FL9, Gag NM10, GagHL9, Pol TF9, PolLF10, and PolTY9) followed by culture for 3 weeks. The cultured cells were analyzed to detect CD8^+^ T cells specific for this cocktail of reported epitope peptides by ICS assay. The CD8^+^ T cells specific for this cocktail were detected in only one individual, VISH421 (Fig. 3A and B). Further analysis demonstrated that the T cells recognized two superimposed epitopes, PolLF10 (LTQIGCTLNF) and PolTF9 (TQIGCTLNF; Fig. 3C). However, VISH421 had HLA-A*02:06, HLA-A*11:01, HLA-B*27:04, HLA-B*40:02, HLA-C*03:04, and HLA-C*12:02, but not HLA-B*15:02. We attempted to identify HLA restriction of these T-cell responses by using cell lines expressing a single HLA allele. The results showed that these T cells recognized only CD4.221-A*02:06 cells prepulsed with PolTF9 peptide (Fig. 3D) and did not recognize CD4.221-B*15:02 cells prepulsed with this peptide (Fig. 3D). This indicated that the responses to PolTF9 peptide were restricted by HLA-A*02:06, rather than HLA-B*15:02. Analysis of epitope peptide titration showed that these T cells weakly recognized CD4.2.221-A*02:06 cells prepulsed with PolTF9 or PolLF10 at 100 nM, but not those at 10 nM (Fig. 3E). These findings suggest that the T-cell response to PolLF10 is also restricted by HLA-A*02:06 and that these HLA-A*02:06-restricted T cells weakly recognize PolLF10/TF9 peptides.

### Recognition of HIV-1-infected cells by HIV-1-specific CD8^+^ T cells elicited in HESN-MSM

**Fig. 4.**
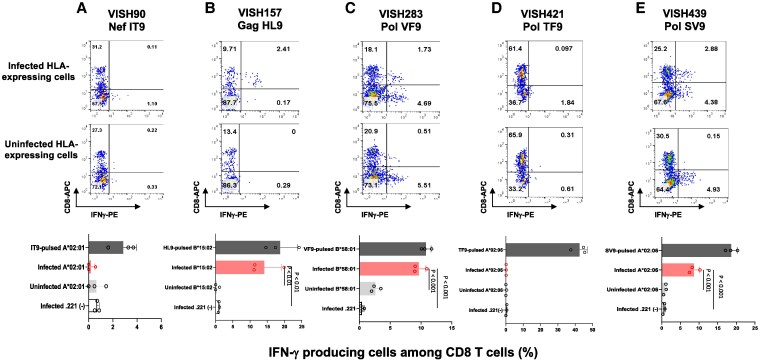
Recognition of HIV-1-infected cells by HIV-1-specific CD8^+^ T cells from five HESN-MSM. Responses of five HIV-1-specific CD8^+^ T cells to HIV-1VI-157X4-infected or uninfected CD4.221 cells expressing the corresponding HLA allele and HIV-1VI-157X4-infected CD4.221 cells were analyzed by ICS assay. HLA-A*02:01-restricted NefIT9-specific T cells from VISH90 (A), HLA-B*15:02-restricted GagHL9-specific T cells from VISH157 (B), HLA-B*58:01-restricted PolVF9-specific T cells from VISH283 (C), HLA-A*02:06-restricted PolTF9-specific T cells from VISH421 (D), and HLA-A*02:06-restricted PolSV9-specific T cells from VISH439 (E) were used as effector T cells. The top two rows display representative data of the responses of HIV-1-specific CD8^+^ T cells to HIV-1-infected and uninfected CD4.221 cells expressing the corresponding HLA allele. The frequencies of p24 antigen-positive cells among HIV-1VI-157X4-infected CD4.221 cells expressing HLA allele and empty CD4.221 cells in each analysis were as follows: A) CD4.221-A*02:01 (66.5%) and CD4.221 cells (37.0%), B) CD4.221-B*15:02 (21.7%) and CD4.221 (41.9%), C) CD4.221-B*58:01 (21.7%) and CD4.221 (6.14%), and D and E) CD4.221-A*02:06 (24.3%) and CD4.221 cells (29.9%). Statistical analysis was performed by unpaired t test. The assays were performed in triplicate.

We investigated the ability of HIV-1-specific CD8^+^ T cells from VISH90, VISH157, VISH283, VISH421, and VISH439 to recognize target cells infected with HIV-1 subtype AE clone VI-157X4, which was previously isolated (31). Identical sequences of these five peptides were detected in the VI-157X4 clone. HIV-1-infected target cells (CD4.221-B*15:02, CD4.221-B*58:01, and CD4.221-A*02:06) were recognized by HIV-1-specific CD8^+^ T cells from VISH157, VISH283, and VISH439, respectively (Fig. 4B, C, and E), whereas two sets of such cells (CD4.221-A*02:01 and CD4.221-A*02:06) were not recognized by HIV-1-specific CD8^+^ T cells from VISH90 and VISH421, respectively (Fig. 4A and D). Thus, two groups of HIV-1-specific CD8^+^ T cells, which can and cannot recognize HIV-1-infected cells, were elicited in HESN-MSM.

### Maintenance of HIV-1-specific memory CD8^+^ T cells in HESN-MSM

**Fig. 5.**
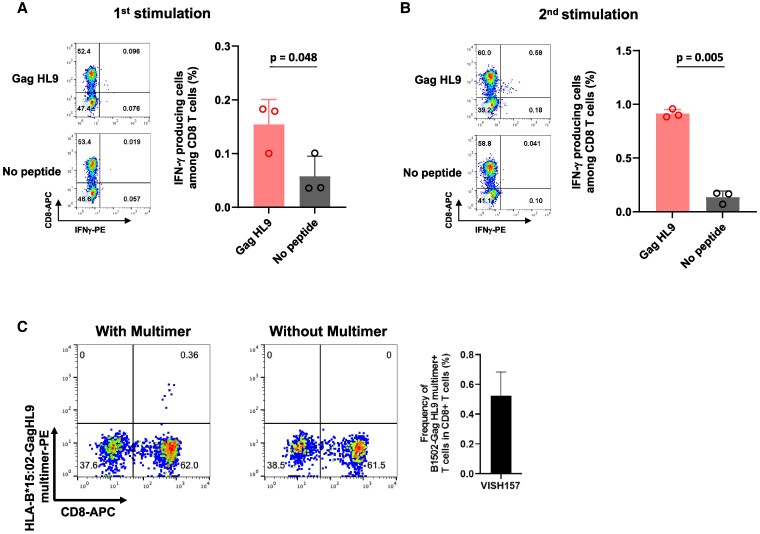
Maintenance of GagHL9-specific memory CD8^+^ T cells in an HLA-B*15:02^+^ HESN-MSM. Responses of bulk T cells derived from VISH157 to CD4.221-B*15:02 prepulsed with GagHL9 peptide were analyzed by ICS assay at the second time point of sample collection (6 months after the initial collection). A) Representative flow cytometry data of bulk T cells cultured for 3 weeks (left) and analysis of triplicate samples (right) are shown. Statistical analysis was performed by unpaired t test. B) The bulk T cells cultured for 3 weeks were re-stimulated with GagHL9 peptide at a concentration of 1 μM and then cultured for 1 week. Responses of these cultured cells to GagHL9 peptide were analyzed by ICS assay. Representative flow cytometry data of bulk T cells cultured for 3 weeks (left) and analysis of triplicate samples (right) are shown. Statistical analysis was performed by unpaired t test. C) Flow cytometric analysis of the bulk T cells using HLA-B*15:02-GagHL9 multimer. Representative data (left) and analysis of triplicate samples (right) are shown.

To investigate the maintenance of CD8^+^ T cells specific for these HIV-1 epitopes, which recognized HIV-1-infected cells, in HESN-MSM, we attempted to collect PBMC samples from three individuals, VISH157, VISH283, and VISH439, at another time point. Specifically, PBMC samples from VISH439, VISH157, and VISH283 were collected at 3, 6, and 19 months after the initial collection, respectively. We stimulated PBMCs from VISH157 with GagHL9 peptide, those from VISH283 with PolVF9 peptide, and those from VISH439 with PolSV9 peptide, and then analyzed the responses of cultured cells to these peptides after culture for 3 weeks. Weak response to GagHL9 peptide was detected in the cultured cells of PBMC samples collected from VISH157 at the second time point (Fig. 5A), whereas T-cell responses to PolVF9 and PolSV9 peptides were not detected in those of VISH283 and VISH439, respectively (Fig. S6). To confirm GagHL9-specific T-cell response, we re-stimulated the cultured cells with GagHL9 peptide and then analyzed the T-cell response to GagHL9 after the cells were cultured for 1 week. GagHL9-specific T-cell response was enhanced after the second stimulation (Fig. 5B). To confirm the existence of GagHL9-specific CD8^+^ T cells among the culture cells, we analyzed these cells using HLA-B*15:02-GagHL9 multimer. The flow cytometric analysis clearly demonstrated the existence of GagHL9-specific CD8^+^ T cells (Fig. 5C). These results together indicate that GagHL9-specific T cells were maintained in VISH157.

### CD8^+^ T-cell responses to two novel HIV-1 epitope peptides in HIV-1 subtype AE-infected individuals

**Fig. 6.**
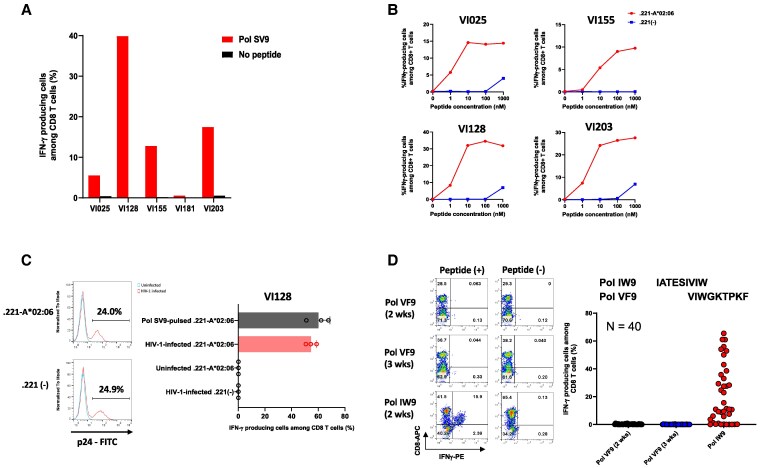
T-cell responses to two novel epitopes, PolSV9 and PolVF9, in HIV-1 subtype AE–infected individuals. A) T-cell responses to PolSV9 peptides in HIV-1 subtype AE–infected HLA-A*02:06^+^ individuals. Responses of bulk T cells derived from five HIV-1 subtype AE–infected HLA-A*02:06^+^ Vietnamese individuals to .221-A*02:06 cells prepulsed with PolSV9 peptide at a concentration of 1 μM were analyzed by ICS assay. B) Responses of bulk T cells in four HIV-1 subtype AE–infected HLA-A*02:06^+^ individuals to CD4.221-A*02:06 cells prepulsed with PolSV9 peptide at concentrations from 1 to 1,000 nM were analyzed by ICS assay. C) Recognition of HIV-1-infected cells by PolSV9-specific CD8^+^ T cells. (Left) Histogram plots display the frequency of p24 antigen-positive cells among VI-157X4-infected CD4.221-A*02:06 cells and VI-157X4-infected CD4.221 cells. (Right) Responses of PolSV9-specific T cells derived from VI-128- to VI-157X4-infected CD4.221-A*02:06 cells, VI-157X4-infected CD4.221 cells, or uninfected CD4.221-A*02:06 cells were analyzed by ICS assay in triplicate. D) T-cell responses to PolVF9 peptides in HIV-1 subtype AE–infected HLA-B*58:01^+^ individuals. PBMCs of 40 HIV-1 subtype AE–infected B*58:01^+^ Vietnamese individuals were stimulated with Pol IW9 or Pol VF9 at a concentration of 1 μM and cultured for 2 or 3 weeks. Responses of PolIW9-specific CD8^+^ T cells (after 2 weeks of culture) and PolVF9-specific CD8^+^ T cells (after 2 and 3 weeks of culture) to CD4.221-B*58:01 prepulsed with the corresponding epitope peptide were analyzed by ICS assay. Representative data of flow cytometric analysis (left) and a summary of analysis in 40 individuals (right) are shown.

A previous study showed that HLA-B*15:02-restricted GagHL9 is the immunodominant epitope in HIV-1 subtype AE–infected Vietnamese (28), while there is no report of HLA-A*02:06-restricted PolSV9-specific and HLA-B*58:01-restricted PolVF9-specific CD8^+^ T cells in such individuals. We therefore investigated whether these T cells are elicited in HIV-1 subtype AE–infected Vietnamese. We stimulated PBMCs from five HIV-1 subtype AE–infected HLA-A*02:06^+^ Vietnamese with PolSV9 peptide and then cultured them for 2 weeks. The cultured cells were analyzed by ICS assay. HLA-A*02:06-restricted PolSV9-specific CD8^+^ T cells were detected in four of five individuals (Fig. 6A and B). These T cells recognized HIV-1 subtype AE–infected cells (Fig. 6C). These findings suggest that PolSV9 is the immunodominant epitope in HIV-1 subtype AE–infected individuals.

We also analyzed HLA-B*58:01-restricted PolVF9-specific CD8^+^ T cells in HIV-1 subtype AE–infected Vietnamese. We stimulated PBMCs from 40 HIV-1 subtype AE–infected HLA-B*58:01^+^ Vietnamese with PolVF9 peptide and then cultured them for 2 or 3 weeks. Interestingly, HLA-B*58:01-restricted PolVF9-specific CD8^+^ T cells were not detected in the cultured cells for 2 or 3 weeks by ICS assay, whereas 31 of these 40 individuals had CD8^+^ T cells specific for a reported HLA-B*58:01-restricted epitope, PolIW9 (32), which overlaps with PolVF9 (Figs. 6D and S7). Thus, PolVF9-specific CD8^+^ T cells were not elicited in HIV-1 subtype AE–infected HLA-B*58:01^+^ Vietnamese individuals who showed strong T-cell responses to PolIW9 epitopes.

## Discussion

We have identified two groups of CD8^+^ T cells in HESN-MSM: one that can recognize HIV-1-infected cells and another that cannot. CD8^+^ T cells specific for NefIT9 and for PolTF9 did not recognize target cells infected with HIV-1 subtype AE. Because NefIT9 and PolTF9 sequences in the VI-157X4 clone were identical to the peptides of these epitopes used in the present study, it is strongly suggested that these epitope peptides are not presented enough for T-cell recognition in HIV-1-infected cells. PolTF9 and NefIT9 were not reported as HLA-A02-restricted HIV-1 epitopes in HIV-1-infected individuals, but in vitro induction of T cells specific for the NefIT9 subtype B consensus sequence (ILDLWVYHT) by HIV-1 peptide-primed dendritic cells was previously reported (29). We investigated whether NefIT9-specific CD8^+^ T cells are elicited in HIV-1 subtype AE–infected HLA-A*02:01^+^ individuals. PBMCs from 10 Vietnamese individuals infected with subtype AE and six Japanese individuals infected with subtype B were stimulated with this peptide and then cultured for 2 weeks. ICS analysis of the cultured cells revealed that CD8^+^ T cells specific for NefIT9 were not detected in the individuals infected with subtype AE but detected in one of the individuals infected with subtype B (Fig. S8). These findings suggest that PolTF9 peptide is not presented by HLA-A*02:06 in HIV-1 subtype AE–infected cells or that the affinity of NefIT9-specific TCR is too low to recognize HIV-1-infected cells. It still remains unclear why CD8^+^ T cells specific for these peptides are elicited in HESN individuals if they are not presented in HIV-1-infected cells. It is hypothesized that these CD8^+^ T cells are elicited by the cross-presentation of exogenous HIV-1 antigens on HLA class I molecules by macrophages or dendritic cells. However, there are several reports of CD8^+^ T cells recognizing different viruses (33–36). Therefore, it is still possible that T cells recognizing these HIV-1 peptides were elicited as T cells cross-reactive with other viruses.

GagHL9-specific CD8^+^ T cells were found in 51 of 83 treatment-naive HLA-B*15:02^+^ Vietnamese individuals infected with subtype AE (28). PolSV9-specific CD8^+^ T cells were found in four of five treatment-naive HLA-A*02:06^+^ individuals infected with subtype AE. Thus, GagHL9 and PolSV9 are the immunodominant epitope in subtype AE infection. A recent study showed that CD8^+^ T cells specific for two HIV-1 immunodominant epitopes were found in HESN-MSM (37). These studies together suggested that CD8^+^ T cells specific for HIV-1 immunodominant epitopes were elicited in HESN-MSM much more than those for subdominant epitopes. Interestingly, GagHL9-specific CD8^+^ T cells were detected in PBMC samples at both the first and the second time points in VISH157, indicating that GagHL9-specific CD8^+^ T cells were maintained for at least 6 months. This suggested that GagHL9-specific memory CD8^+^ T cells were maintained in this individual. GagHL9 and GagHL9-2I sequences were found in 77 and 10% of subtype AE–infected Vietnamese individuals, respectively, while GagHL9-specific CD8^+^ T cells recognized GagHL9 and GagHL9-2I at equivalent rates (28). These findings imply that GagHL9-specific memory CD8^+^ T cells were maintained by repeated exposure to subtype AE virus circulating in Vietnam.

We did not clarify whether HIV-1-specific CD8^+^ T cells were not elicited from PBMCs from healthy individuals who had not been exposed to HIV-1 in the present study because we do not have a cohort of >300 Vietnamese individuals unexposed to HIV-1. Approximately half of previous studies of HIV-1-specific T cells in HESN individuals analyzed the induction of HIV-1-specific T cells in only a small number of individuals unexposed to HIV-1 but did not show a definitive response specific for HIV-1 because ELISpot assay was used in these studies (7, 11, 14, 15, 27). Previous studies demonstrated that HIV-1-specific CD8^+^ T cells were detected at a frequency of 1 per 0.3–1 × 10^6^ CD8^+^ T cells in healthy individuals who had not been infected with or exposed to HIV-1 (19). In the present study, we used ∼5 × 10^5^ PBMCs to induce CD8^+^ T cells specific for each set of pooled peptides. Because 5 × 10^5^ PBMCs contain 1–1.5 × 10^5^ CD8^+^ T cells, it is assumed that less than one HIV-1-specific CD8^+^ T cell is included among 5 × 10^5^ PBMCs from individuals who have not been exposed to HIV-1. In addition, it is difficult to expand HIV-1-specific CD8^+^ T cells from naive T cells by using HIV-1 peptide alone. The in vitro induction of HIV-1-specific CD8^+^ T cells from naive T cells was reported only when naive T cells were stimulated with HIV-1 peptides and some ligands such as STING ligand or lipopolysaccharide (LPS) (38, 39) or with HIV-1 peptide-pulsed dendritic cells (29). We therefore assume that HIV-1-specific CD8^+^ T cells in HESN-MSM are derived from memory CD8^+^ T cells, but not from naive T cells. The present study has a limitation in that we did not show that HIV-1-specific CD8^+^ T cells were not elicited from PBMCs from a large number of individuals who had not been exposed to HIV-1. A further study of HIV-1-specific CD8^+^ T cells in both HESN-MSM and healthy individuals unexposed to HIV-1 is expected to clarify whether these T cells are elicited from memory T cells in HESN-MSM.

HLA-B*58:01-restricted PolVF9-specific CD8^+^ T cells, which were elicited in an HLA-B*58:01^+^ HESN-MSM, recognized HIV-1-infected target cells, indicating that PolVF9 is presented in HIV-1-infected cells. In contrast, PolVF9-specific CD8^+^ T cells were not detected in 40 HLA-B*58:01^+^ individuals chronically infected with HIV-1 subtype AE. This suggests that PolVF9 is very rarely recognized as a subdominant epitope or is not an epitope in the chronic phase of HIV-1 infection. Because ∼85% of circulating HIV-1 subtype AE in our Vietnamese cohort harbored the same sequence as PolVF9 (VIWGKTPKF; Fig. S9) (40), it is not the case that the accumulation of mutations in PolVF9 affects the induction of PolVF9-specific T cells. One possible explanation for this is that PolVF9-specific CD8^+^ T cells were elicited in the acute infection phase, but disappeared in the chronic infection phase. Thus, the role of PolVF9-specific CD8^+^ T cells in HIV-1 infection remains unclear. Further analysis of PolVF9-specific CD8^+^ T cells may be useful to elucidate T-cell immunity against HIV-1 before and in the acute phase of HIV-1 infection.

Compared with HIV-1-infected individuals, HESN-MSM had far fewer HIV-1-specific CD8^+^ T cells, and the responses of these T cells to the epitope peptides were weaker. Therefore, we may not have detected HIV-1-specific CD8^+^ T cells in some cases in which these T cells were very weakly elicited. Assays using HLA tetramers or HLA multimers may help to identify HIV-1-specific T cells or to confirm their existence. In the present study, we used HLA-A*02:06 and HLA-B*15:02 multimers and clearly demonstrated the existence of GagHL9- or PolSV9-specific T cells that recognized HIV-1-infected cells. However, because a positive control of PolVF9-specific T cells derived from HIV-1-infected individuals and stock of the bulk cultured T cells containing PolVF9-specific T cells were not available, we could not identify PolVF9-specific T cells using HLA-B*58:01-PolVF9 multimer.

In the present study, we identified HIV-1-specific CD8^+^ T-cell responses in eight of 370 HESN-MSM and identified HLA-restricted CD8^+^ T cells specific for five HIV-1 peptides. We previously demonstrated that HIV-1-specific CD8^+^ T-cell responses were detected in only 2% of Japanese HESN-MSM (37), suggesting together with the present study that HIV-1-specific CD8^+^ T-cell responses are detected in ∼2% of HESN-MSM in Asia. This frequency was much less than that in previous studies of HESN-sexual workers (7, 8, 11) and HESN-MSM (17, 25). It remains unclear why this difference in HIV-1-specific CD8^+^ T-cell responses was found between two HESN cohorts in Asia and other HESN cohorts, but this may be due to different assays used to detect HIV-1-specific T cells or differences in the degree of exposure to HIV-1. A large-scale and careful analysis of HIV-1-specific T cells in HESN using multiple assays to detect HIV-1-specific T cells is expected to elucidate the mechanism of induction of HIV-1-specifc T cells in HESN.

In the present study, two groups of HIV-1-specific CD8^+^ T cells, which can and cannot recognize HIV-1-infected cells, were elicited in HESN-MSM. HIV-1-specific CD8^+^ T cells that can recognize HIV-1-infected cells were composed of CD8^+^ T cells specific for the immunodominant or nonimmunodominant HIV-1 epitope. We have clearly demonstrated the existence of HIV-1-specific CD8^+^ T cells in some HESN-MSM, suggesting that HIV-1-specific CD8^+^ T cells can be elicited before HIV-1 infection is established.

## Materials and methods

### Subjects and ethics statements

All 370 HESN-MSM were recruited at the Sexual Health Promotion clinics in Hanoi Medical University (HMU) hospitals, Hanoi, Vietnam. The study protocol was approved by the ethics committee of Kumamoto University (Rinri2289) and HMU (364/GCN-HDDDNCYSH-DHYHN). Informed consent was obtained from all HESN-MSM, in accordance with the tenets of the Declaration of Helsinki. The inclusion criteria of HESN individuals in the MSM cohort were as follows: (1) >18 years old, (2) reporting anal sex (insertive or receptive) with men in the previous 6 months, and (3) HIV-1-specific antibody-negative and HIV-1 p24 antigen-negative results just before peripheral blood collection. In addition, we recruited five HLA-A*02:06^+^ and 42 HLA-B*58:01^+^ treatment-naive Vietnamese individuals chronically infected with HIV-1 subtype AE in the National Hospital of Tropical Diseases, Hanoi, Vietnam. The study protocol was approved by the Ethics Committee of the Vietnamese Ministry of Health (1666/Q-D-BYT) and by the Ethics Committee of Kumamoto University (RINRI-1340 and GENOME-342). Informed consent was obtained from these individuals in accordance with the tenets of the Declaration of Helsinki.

### HIV-1-negative status of participants

The HIV-1-negative status of all participants was confirmed by using the HIV-1/2 Ag/Ab COMBO (Abbott, USA) just before the collection of peripheral blood used in this study. The HIV-1-negative status of six individuals (VISH157, VISH257, VISH283, VISH288, VISH418, and VISH439) with HIV-1-specific T cells was further confirmed between 1 year and 10 months and 3 years and 8 months after the first collection of peripheral blood in which HIV-1-specific T cells were detected.

### HLA genotyping

We identified HLA class I genotypes of individuals in whom HIV-1-specific CD8^+^ T cells were detected by using the Luminex microbead method at the NPO HLA Laboratory (Kyoto, Japan).

### Peptides

Two hundred and eighty-one 17-mer overlapping peptides spanning Nef, Gag, and Pol of HIV-1 subtype AE consensus sequences and 8- to 11-mer truncated peptides as well as truncated peptides of some 17-mer peptides were synthesized using an automated multiple-peptide synthesizer and purified by high-performance lipid chromatography (HPLC). These 17-mer peptides overlapped by 11 amino acids. Thirty-five peptide cocktails were generated by mixing eight or nine peptides per cocktail (four for Nef, 10 for Gag, and 21 for Pol). Subsequently, six peptide pools were made as follows: pool 1: four Nef cocktails containing 33 Nef 17-mers, pool 2: five Gag cocktails containing 40 Gag 17-mers, pool 3: five Gag cocktails containing 42 Gag 17-mers, pool 4: seven Pol cocktails containing 56 Pol 17-mers, pool 5: seven Pol cocktails containing 56 Pol 17-mers, and pool 6: seven Pol cocktails containing 54 Pol 17-mers. The purity was examined using HPLC and mass spectrometry. Only high-purity peptides (>90%) were used in the study.

### Cell lines

CD4.221 cells and transfected with a single HLA-A allele (A*11:01, A*11:02, A*02:01, A*02:03, A*33:03, A*24:02, or A*02:06), a single HLA-B allele (B*15:02, B*15:25, B*58:01, B*35:05, or B*46:01), and a single HLA-C allele (C*03:04, C*08:01, C*03:02, C*04:01, C*04:03, C*07:02, or C*01:02) were previously generated (28, 41–43). C1R cells expressing HLA-B*15:01 were previously described (43). All cell lines were maintained in RPMI 1640 medium (Thermo Fisher Scientific, Waltham, MA, USA) containing 5% fetal bovine serum (FBS) and 0.15 mg/mL hygromycin B (Merck Millipore, Burlington, MA, USA) or 0.2 mg/mL of neomycin (Thermo Fisher Scientific).

### HIV-1 subtype AE clone

An infectious provirus HIV-1 subtype AE, VI-157X4, was previously generated from an HIV-1-infected Vietnamese individual (31).

### Expansion of HIV-1-specific bulk T cells and ICS assay

A total of 3 × 10^5^ to 5 × 10^5^ PBMCs from HESN-MSM were stimulated with each peptide pool at a concentration of 5 μM or single peptides at a concentration of 1 μM, followed by culture for 3 weeks in RPMI1640 medium (Thermo Fisher Scientific) containing 10% FBS, 10 ng/mL human rIL-2 (Pepro Tech, Cranbury, NJ, USA), ×1 minimum essential medium (MEM) nonessential amino acids solution (Thermo Fisher Scientific), and 1 mM sodium pyruvate solution (Thermo Fisher Scientific). Cultured bulk T cells (3,000 or 5,000 cells) were co-cultured with peptide pool, HLA-expressing CD4.221 cells or C1R cells (15,000 or 25,000 cells) prepulsed with peptides in a 96-well plate for 4 h at 37 °C with 10 g/mL brefeldin A (Sigma-Aldrich, St Louis, MO, USA). The cells were then fixed with 4% paraformaldehyde (BD Biosciences, La Jolla, CA, USA) and permeabilized with saponin buffer (0.1% Saponin/5% FBS/phosphate-buffered saline) after staining with allophycocyanin (APC)-labeled CD8-specific mAb (BioLegend, San Diego, CA, USA). Subsequently, the cells were stained with phycoerythrin-labeled gamma interferon-specific mAb (BioLegend). The percentage of IFN-γ-producing cells among CD8^+^ T cells was analyzed by FACS Canto II (BD Biosciences) and analyzed using FlowJo 10.7.1 software.

The relative frequency of IFN-γ^+^CD8^+^ cells was calculated as the frequency of IFN-γ^+^CD8^+^ cells among CD8^+^ cells stimulated with peptides minus that of IFN-γ^+^CD8^+^ cells among CD8^+^ cells without peptides. For the screening using peptide pool, >0.4% frequency of IFN-γ^+^CD8^+^ cells among total CD8^+^ cells was selected to indicate potential positive responses, while cases with a high background level that showed a high frequency of IFN-γ^+^CD8^+^ cells in samples without peptides (>0.5%) were excluded. Positive responses were further confirmed by a triplicate assay.

### Degranulation assay

CD4.221-B*15:02 cells prepulsed with GagHL9 or CD4.221-A*02:06 cells prepulsed with PolSV9 were used as stimulator cells. These cells were added to a 96-well plate together with the cultured bulk T cells, and the cells were incubated for 4 h at 37 °C with APC-labeled anti-CD107a (LAMP-1) mAb (BioLegend). The cells were then stained with Pacific blue-labeled anti-CD3 mAb (BioLegend), PerCP-Cyanine5.5-labeled anti-CD8 mAb (BioLegend), and LIVE/DEAD Fixable Near-IR Dead Cell Stain Kit (Invitrogen). Staining data were acquired on a FACSCanto II (BD Biosciences) and analyzed using FlowJo 10.7.1 software.

### Staining of GagHL9-specific and PolSV9-specific CD8^+^ T cells by multimers

HLA-B*15:02-GagHL9 and HLA-A*02:06-PolSV9 dextramers were synthesized using U-Load Dextramer Kit MHC I HLA-B*1502 and HLA-A*0206 (Immudex, Copenhagen, Denmark). Bulk T cells were suspended in 0.53 nM dextramer in RPMI 1640 supplemented with 10% fetal calf serum (FCS) (R10) and then incubated at 37 °C for 30 min. The cells were then washed twice with R10, followed by staining with fluorescein isothiocyanate (FITC)-labeled anti-CD3 mAb (BioLegend), APC-labeled anti-CD8 mAb (BioLegend), and LIVE/DEAD Fixable Near-IR Dead Cell Stain Kit (Invitrogen) at 4 °C for 30 min. The cells were washed twice with R10. Data were analyzed with a FACS Canto II (BD Biosciences). FlowJo 10.7.1 software was used for the analyses.

### Recognition of HIV-1-infected cells by HIV-1-specific CD8^+^ T cells

CD4.221 cells or CD4.221 cells expressing a single HLA were infected with HIV-1 subtype AE clone VI-157X4 for 3 or 4 days. The frequency of HIV-1-infected cells was determined by intracellular staining with FITC-conjugated anti-HIV-1 p24 mAb, KC-57 (Beckman Coulter, Brea, CA, USA). The HIV-1-infected cells were cocultured with HIV-1-specific bulk T cells in a 96-well plate for 4 h at 37 °C, and the percentage of IFN-γ-producing cells among the CD8^+^ T-cell population was analyzed by the ICS assay described above.

### Statistical analysis

Unpaired t test was performed to compare two groups in this study. A *P*-value of <0.05 was considered statistically significant. Graphs were created in GraphPad Prism 8.4.3.

## Supplementary Material

pgaf336_Supplementary_Data

## Data Availability

The datasets analyzed during the current study are presented in all figures and in the Supplementary material.
