# Supplementary material for: HIV-1-specific CD8+ T cells with different abilities to recognize HIV-1-infected cells in HIV-1-exposed seronegative individuals
Source: PNAS Nexus. 2025 Oct 22;4(11):pgaf336. doi: 10.1093/pnasnexus/pgaf336 (PMC12598662; doi:10.1093/pnasnexus/pgaf336)
Supplement: pgaf336_Supplementary_Data [file pgaf336_supplementary_data.pdf]

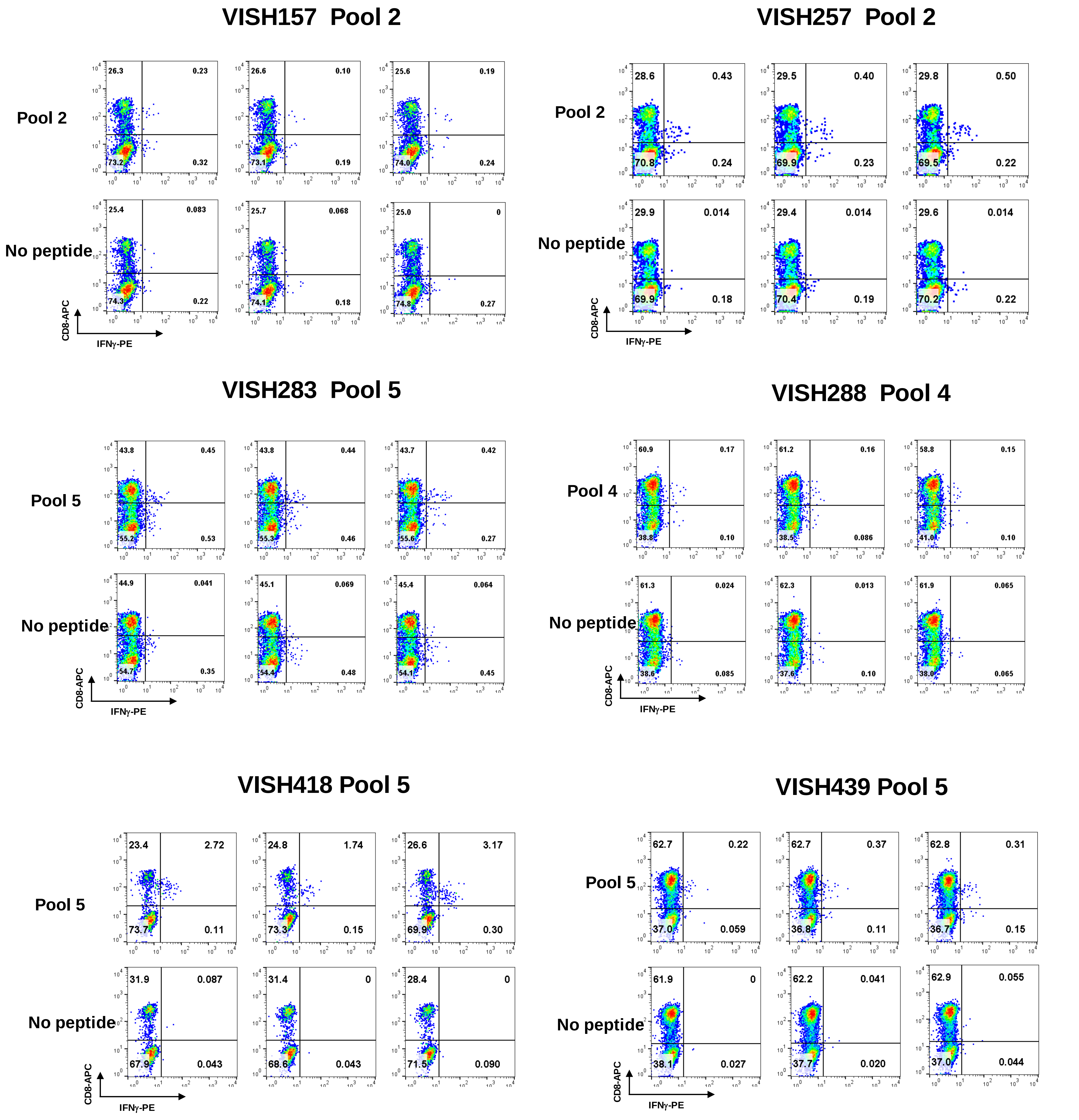

Figure S1. Flow cytometry analysis of $CD8^+$ T cell responses to HIV-1subtype AE pool peptides in six HESN-MSM

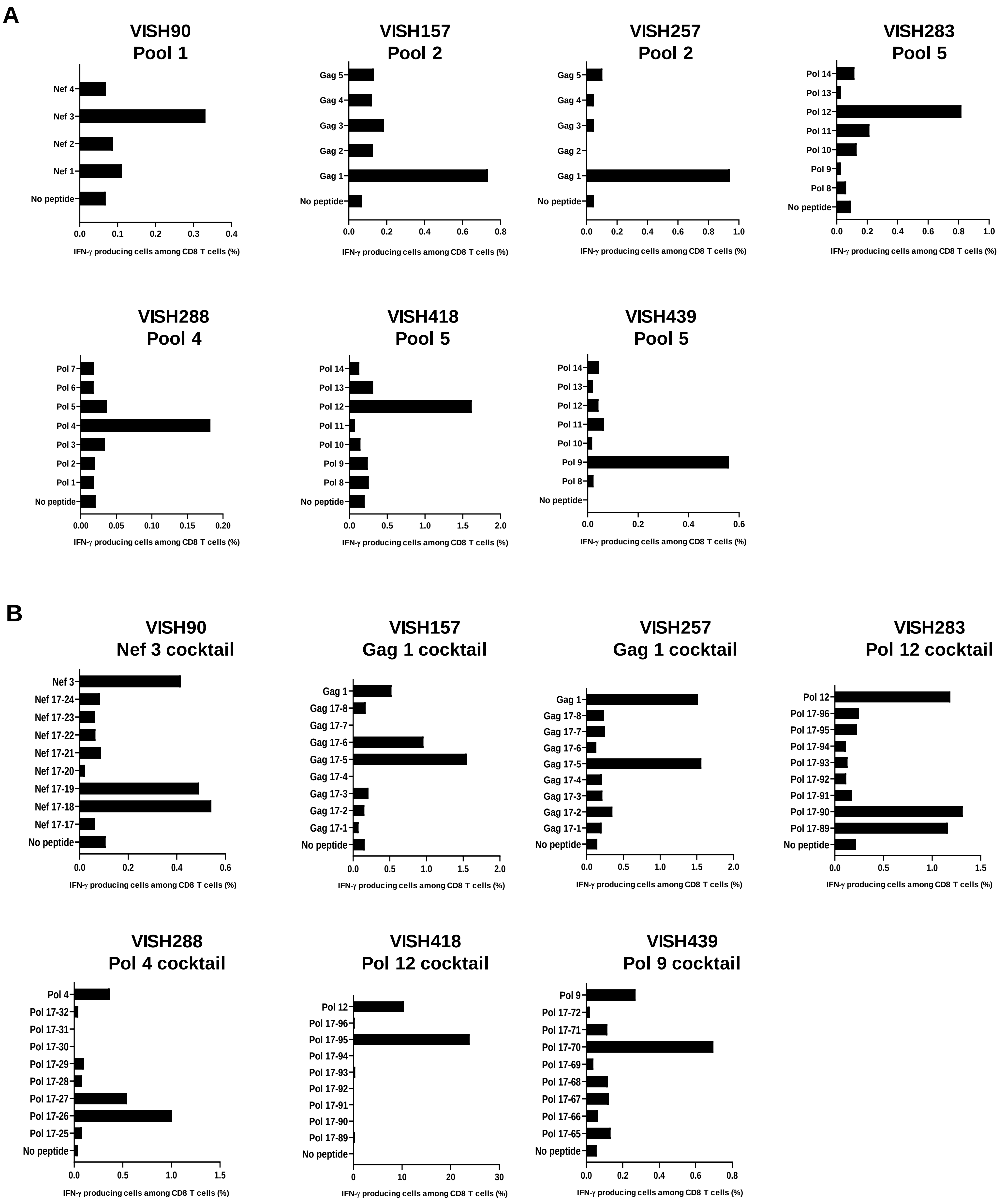

Figure S2. Identification of CD8$^+$ T cells specific for single 17-mer peptides in seven HESN-MSM

A. HIV-1-specific CD8$^+$ T-cell responses to peptide cocktail. Responses of bulk T cells derived from seven HESN-MSM to each peptide cocktail at a concentration of 1 μM were analyzed by ICS assay. **B.** HIV-1-specific CD8$^+$ T-cell responses to single 17-mer overlapping peptides. Responses of bulk T cells derived from seven HESN-MSM to each 17-mer peptide at a concentration of 1 μM were analyzed by ICS assay.

Nef 3 cocktail	
Nef 17-24	FGWCFKLVPVDPREVEE
Nef 17-23	IRYPLCFGWCFKLVPVD
Nef 17-22	YTPGPGIRYPLCFGWCF
Nef 17-21	FPDWQNYTPGPGIRYPL
Nef 17-20	YNTQGFFPDWQNYTPGP
Nef 17-19	ILDLWVYNTQGFFPDWQ
Nef 17-18	SKKRQEILDLWVYNTQG
Nef 17-17	LDGLIYSKKRQEILDLW

Gag 1 cocktail	
Gag 17-8	RFALNPGLLETAEGCQQ
Gag 17-7	ASRELERFALNPGLLET
Gag 17-6	MKHLVWASRELERFALN
Gag 17-5	GKKKYRMKHLVWASREL
Gag 17-4	IRLRPGGKKKYRMKHLV
Gag 17-3	LDAWEKIRLRPGGKKKY
Gag 17-2	VLSGGKLDAWEKIRLRP
Gag 17-1	MGARASVLSGGKLDAWE

Pol 4 cocktail	
Pol 17-32	KALTEICKEMEKEGKIS
Pol 17-31	LTEEKIKALTEICKEME
Pol 17-30	KVKQWPLTEEKIKALTE
Pol 17-29	PGMDGPKVKQWPLTEEK
Pol 17-28	VPVTLKPGMDGPKVKQW
Pol 17-27	ISPIDTVPVTLKPGMDG
Pol 17-26	CTLNFPISPIDTVPVTL
Pol 17-25	MLTQIGCTLNFPISPID

Pol 9 cocktail	
Pol 17-72	FVNTPPLVKLWYQLEKD
Pol 17-71	WIPEWEFVNTPPLVKLW
Pol 17-70	EYWQATWIPEWEFVNTP
Pol 17-69	WETWWMEYWQATWIPEW
Pol 17-68	PIQKETWETWWMEYWQA
Pol 17-67	TPKFRLPIQKETWETWW
Pol 17-66	IVIWGKTPKFRLPIQKE
Pol 17-65	MGYELHPDRWTVQPIEL

Pol 12 cocktail	
Pol 17-96	FVNTPPLVKLWYQLEKD
Pol 17-95	WIPEWEFVNTPPLVKLW
Pol 17-94	EYWQATWIPEWEFVNTP
Pol 17-93	WETWWMEYWQATWIPEW
Pol 17-92	PIQKETWETWWMEYWQA
Pol 17-91	TPKFRLPIQKETWETWW
Pol 17-90	IVIWGKTPKFRLPIQKE
Pol 17-89	KIATESIVIWGKTPKFR

Figure S3. List of 17-mer peptides in positive Pool peptide cocktail

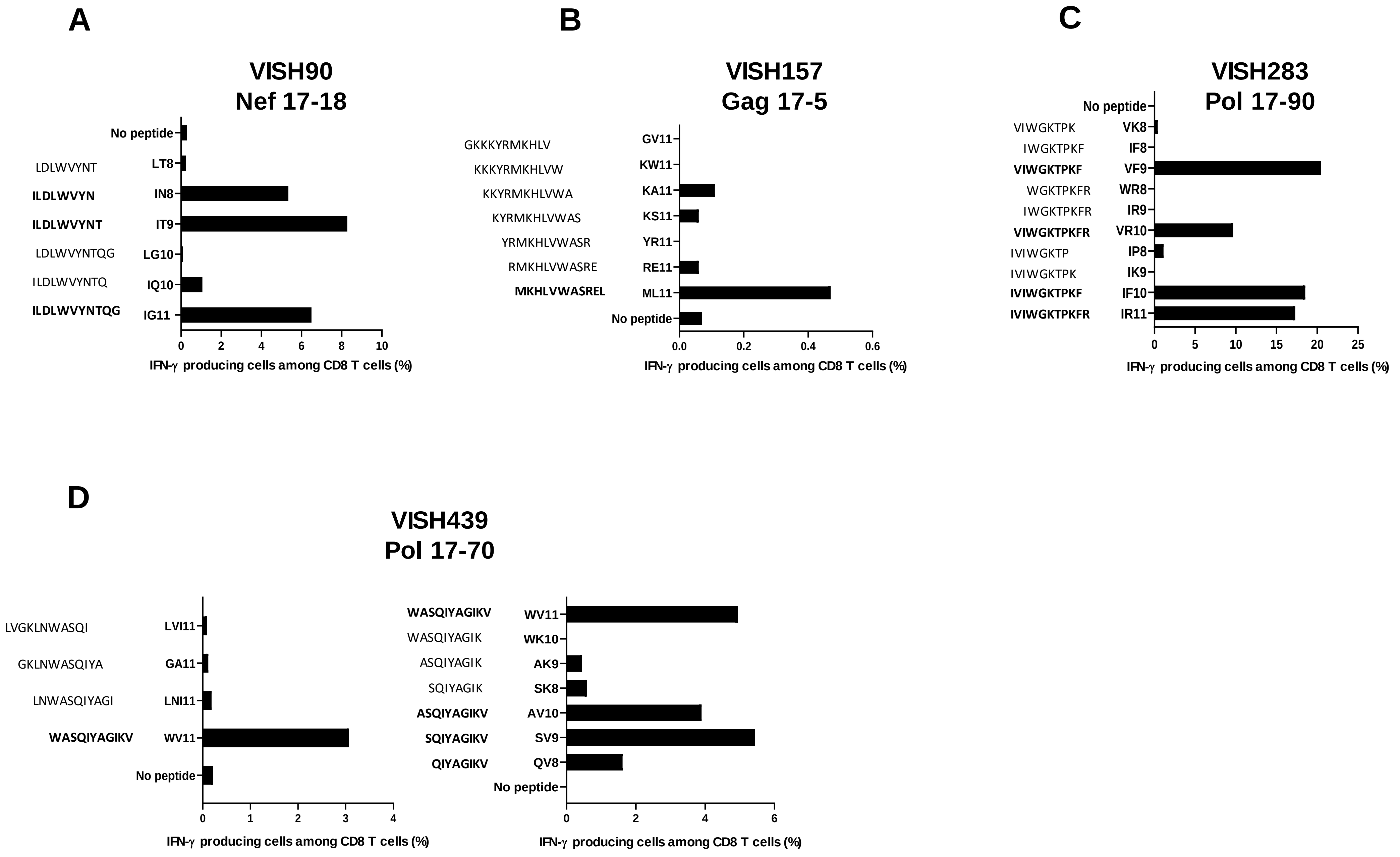

Figure S4. Responses of 17-mer-specific CD8⁺ T cells derived from four individuals to truncated HIV-1 peptides

A. Responses of Nef17-18-specific CD8$^+$ T cells derived from VISH90 to CD4.221-A*02:01 prepulsed with truncated peptides of NefIG11 at a concentration of 1 μM. **B.** Responses of Gag 17-5-specific CD8$^+$ T cells derived from VISH157 to CD4.221-B*15:02 prepulsed with 11-mer peptides spanning Gag 17-5 peptide at a concentration of 1 μM. **C.** Responses of Pol 17-90-specific CD8$^+$ T cells derived from VISH283 to CD4.221-B*58:01 prepulsed with truncated peptides spanning PolIR11 peptide at a concentration of 1 μM. **D.** (Upper) Responses of Pol 17-70-specific CD8$^+$ T cells from VISH439 to CD4.221-A*02:06 cells prepulsed with four 11-mer peptides spanning Pol 17-70 peptide at a concentration of 1 μM. (Lower) Responses of Pol 17-70-specific CD8$^+$ T cells to CD4.221-A*02:06 cells prepulsed with truncated peptides spanning Pol WV11 peptide at a concentration of 1 μM.

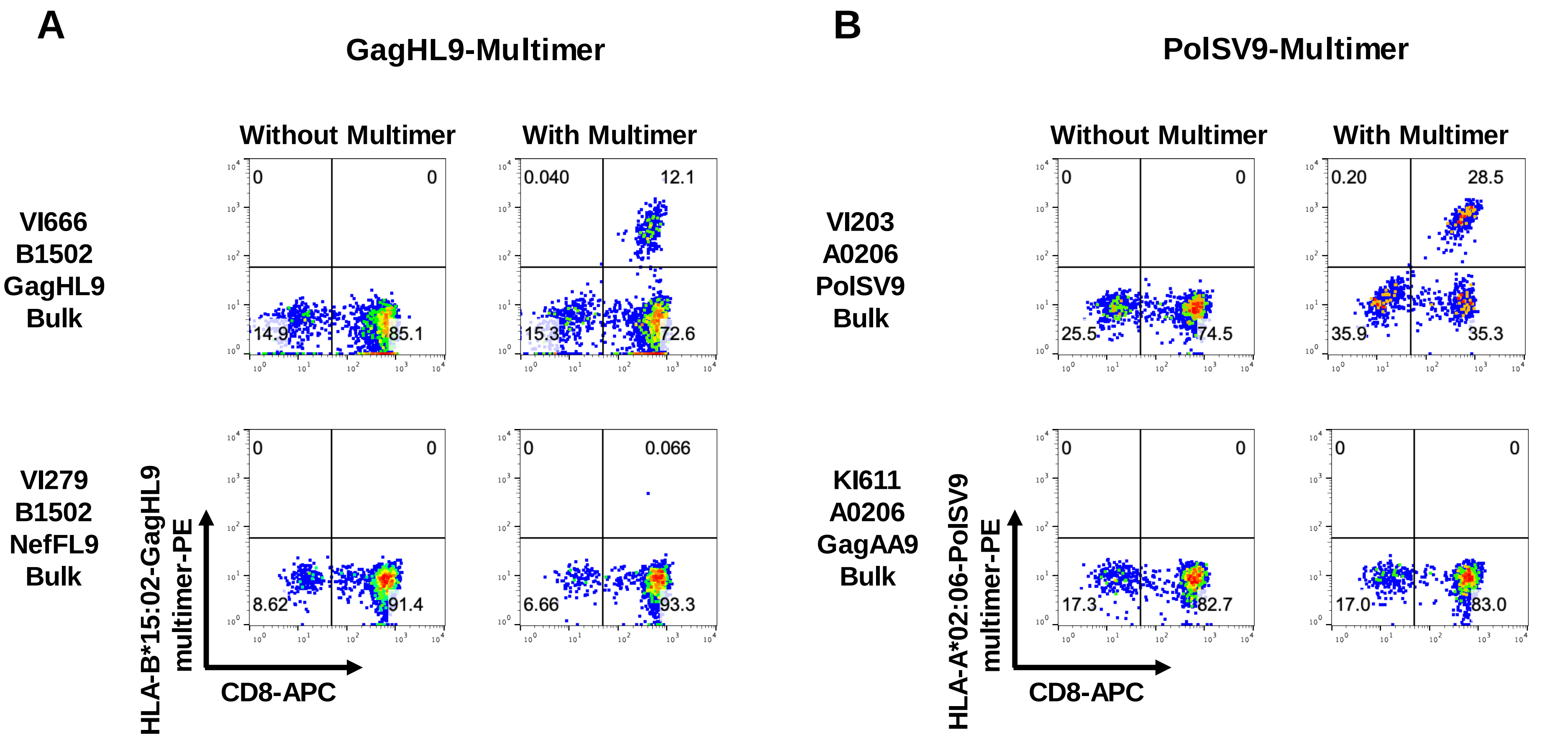

Figure S5. Specificity of HLA-B*15:02-GagHL9 multimer and HLA-A*02:06-PolSV9 multimer

A. Staining of GagHL9 or NefFL9 peptide specific bulk T cell established from HIV-1-infected HLA-B*15:02+ individuals (VI666 and VI279) by PE-conjugated HLA-B*15:02-GagHL9 multimer (Dextramer). **B.** Staining of PolSV9 or GagAA9 peptide specific bulk T cell established from HIV-1-infected HLA-A*02:06+ individuals (VI203 and KI611) by PE-conjugated HLA-A*02:06-PolSV9 multimer (Dextramer).

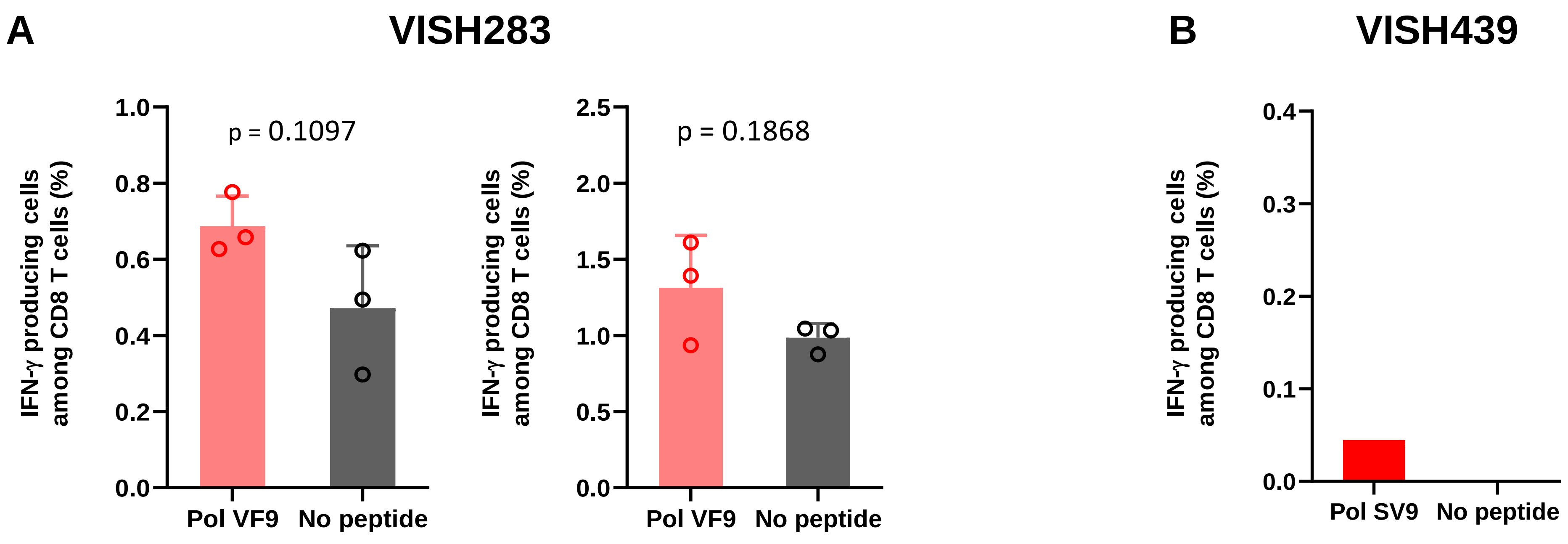

Figure S6. **Pol VF9- specific CD8+ T cells and SV9-specific CD8+ T cells in HESN-MSM**

A. Responses of bulk T cells derived from VISH283 to CD4.221-B*58:01 prepulsed with PolVF9 peptide were analyzed by ICS assay at the 2nd timepoint of sample collection (19 months after the initial collection). Left) Bulk T cells were cultured for 3 weeks and analysis of triplicate samples are shown. Right) The bulk T cells cultured for 3 weeks were re-stimulated with PolVF9 peptide at a concentration of 1 μM and then cultured for 1 week. Analysis of triplicate samples are shown. Statistical analysis was performed by unpaired t test. **B**. Responses of bulk T cells derived from VISH439 to CD4.221-A*02:06 prepulsed with PolSV9 peptide was analyzed by ICS assay at the 2nd timepoint of sample collection (3 months after the initial collection).

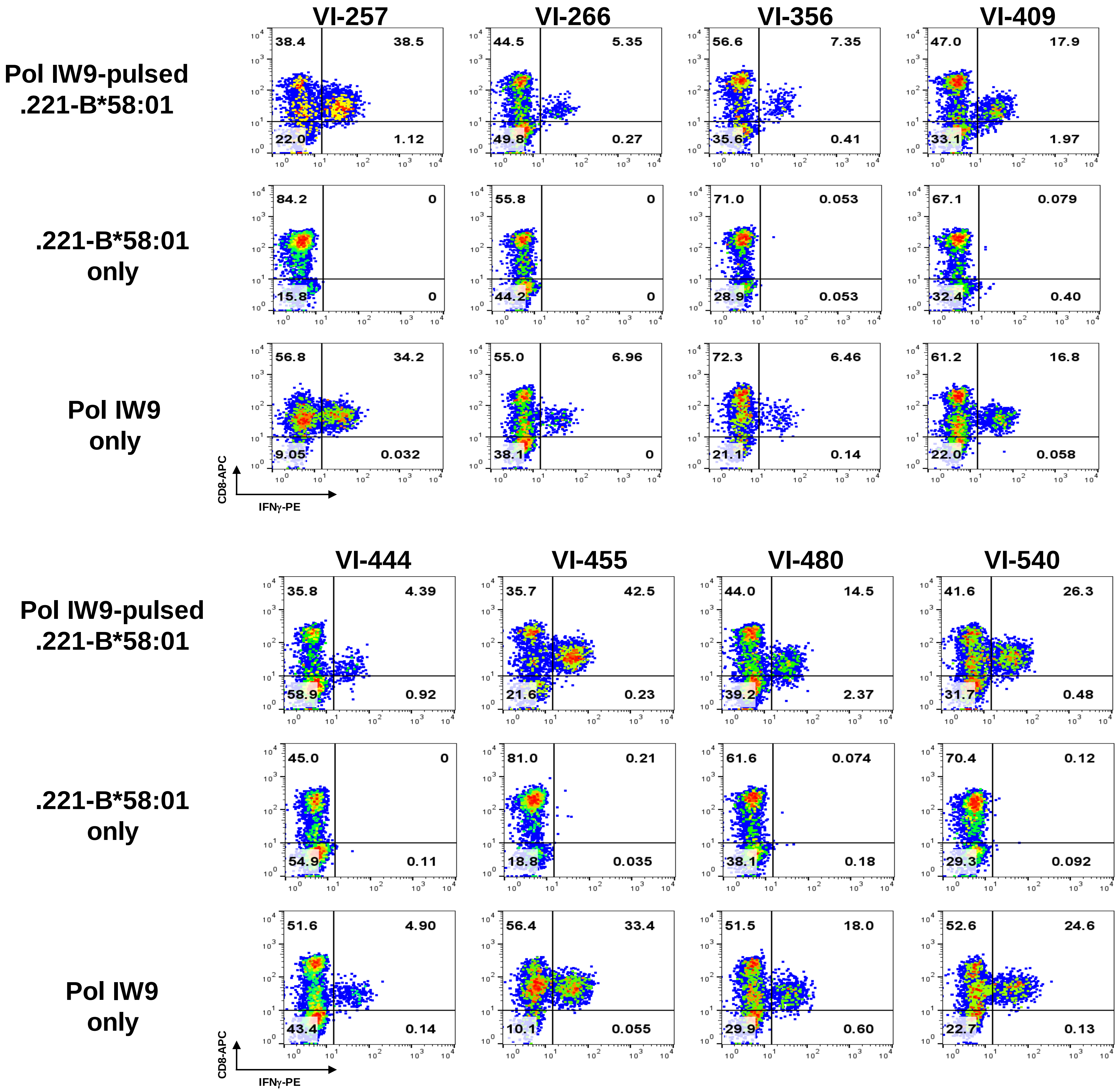

Figure S7. Flow cytometry analysis of CD8^{+} T cell responses to Pol IW9 peptide in eight representative responders infected with HIV-1.

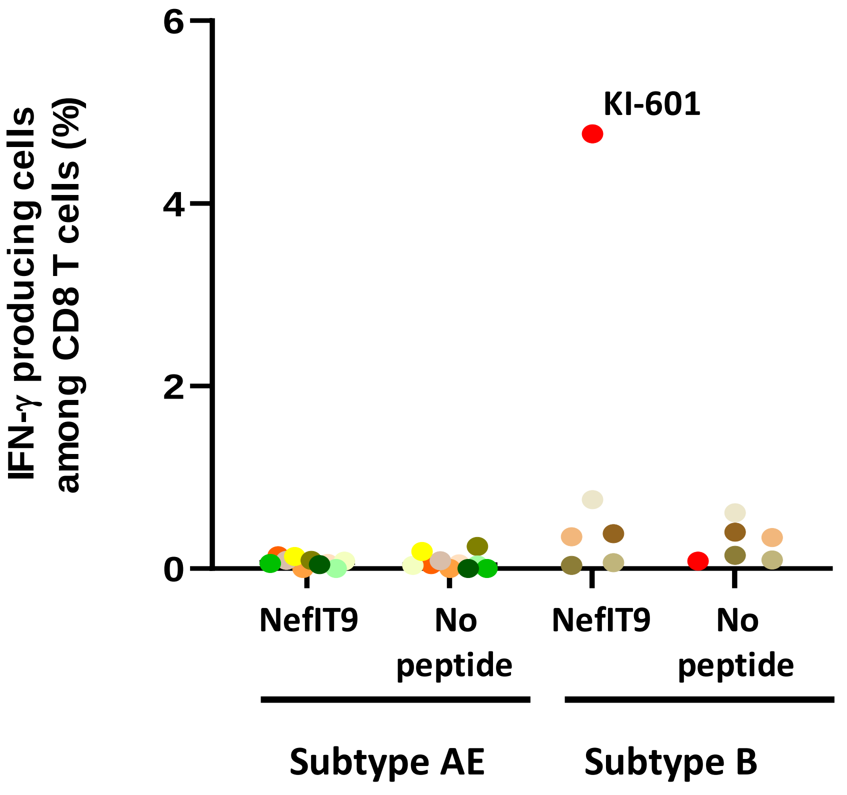

Figure S8. T cells responses to NefIT9 in HIV-1 subtype AE-infected individuals and HIV-1 subtype B-infected individuals.

Responses of bulk T cells derived from 10 HIV-1 subtype AE-infected HLA-A*02:01+ individuals and 6 HIV-1 subtype B-infected HLA-A*02:01+ individuals to CD4.221-A*02:01 prepulsed with NefIT9 peptide at a concentration of 1 μM were analyzed by ICS assay. Each dot represented one individual.

Epitope	HXB2 position	Sequence	No. positive/Total No.	Frequency (%)
Pol VF9	536-574	VIWGKTPKF	304/359	84.7
		IIWGKTPKF	19/359	5.3

Figure S9. Frequency of HIV-1 sequences corresponding to PolVF9 among Vietnamese individuals chronically infected with HIV-1 subtype AE.

Sequences data of Pol region including Pol 536-574 were deposited in the DDBJ/EMBL/GenBank under accession numbers LC100902 to LC101260.
